# The Royal Army Medical Corps

**Published:** 1901-11-09

**Authors:** 


### The Hospital

A Journal of

The Medical Sciences and Hospital Administration.

Vol. XXXI.—No. 789.] Edited by Sir Henry Burdett, K.C.B., and Solomon C. Smith, M.D., M.R.C.P. [November 9, 1901.

### The Royal Army Medical Corps.

Lord Beaconsfield once extinguished a second-class politician among his contemporaries by describing him in the House of Commons as "a superior person," and Sir Frederic Treves has lately been doing good service in a letter to the *Times*, in which he has dealt at some length with the objections raised by critics of the "superior" class against the proposals of Mr. Brodrick's Committee with regard to the reorganisation of the Royal Army Medical Corps. The public and the profession are, we think, alike indebted to Sir Frederic for his timely defence of the committee's proposals, and for his clear version of what it is that they are likely and are intended to effect. It is a matter of primary concern to the public, more especially in view of the increased interest in military matters which has been awakened by the war, that the Army should be provided with a thoroughly efficient medical and surgical staff; while it is of no less concern to the profession, looking at the subject even from the narrowest possible point of view, that the medical department of the Army, under whatever name it may be called, should be so organised as to offer a satisfactory career to capable and self-respecting men, and that it should attract to its ranks for the future candidates worthy of its high position in the past. A good deal of the business of the War Office, unless that wonderful machine is much belied by public opinion, is so conducted that aspirants for combatant military employment are too often compelled

"to wait;"

"While ladies interpose, and *clerks* debate;"

and it should be a matter for no small congratulation to the medical profession that the representative of the Office in the House of Commons should himself have made an earnest endeavour to take the bull of medical discontent by the horns, and to find a way of satisfying at the same time the legitimate requirements of Army surgeons and the legitimate requirements of the public and of the service. The results of such an endeavour should not be looked at with a manifest desire to pick holes in them; and, although they may quite rightly be submitted to criticism, it should be to criticism of a sympathetic and not of a purely antagonistic character.

A point on which much stress has been laid by the critics, and on which Sir Frederick Treves defends the recommendations of the Committee, is the one additional examination which officers of considerable standing in the service will be called upon to pass as a condition antecedent to promotion to certain higher grades; and his defence is the very reasonable one that this examination will be strictly confined to a knowledge of laws and regulations which officers in those higher grades will be called upon to enforce. With one exception, to which we will revert, the examinations which form part of the scheme seem to us to be merely of such a nature as to render certain the possession of knowledge which may not of necessity be implied by the holding of a medical qualification, and which, for some reason or another, is certain, or highly probable, to be required from an officer of the corps. Many matters, the character of a suggested water-supply for example, which a private practitioner would at once refer to an expert, must be determined by the Army surgeon upon his own responsibility; and, until some better method of testing knowledge than by examination has been discovered, we hardly see how this method can either be dispensed with, or properly regarded as a grievance by those who are called upon to submit to it. The one exception which we should be inclined to take, and as to which we think the provisions of the scheme might be reconsidered with advantage, has reference to the entrance examination for candidates of a certain class. For the holders of what are called the higher qualifications, an entrance examination is at once an absurdity and a humiliation. A candidate who was a Fellow of the Royal College of Surgeons of England, and a graduate of a distinguished University (and these are the sort of men whom it is desirable to attract to the Army Medical Corps) would usually be quite capable of examining his examiners, and might even find good ground for rejecting them.

It seems certain that men possessing such qualifications should be exempt from the entrance examination; and the only objection to such exemption, namely, that it would be difficult for the War Office to draw the line between these qualifications and others, would at once be met by deputing the task to the General Medical Council. We would go farther, and would urge that men possessing the accepted qualifications should from the first receive a higher pay than candidates only admissible through examination; inasmuch as such an arrangement would be a merely honest recognition of their greater value in the professional market.

